# Advances of research in diabetic cardiomyopathy: diagnosis and the emerging application of sequencing

**DOI:** 10.3389/fcvm.2024.1501735

**Published:** 2025-01-13

**Authors:** Qianqian He, Ze Lai, Zhengyao Zhai, Beibei Zou, Yangkai Shi, Chao Feng

**Affiliations:** ^1^ Department of Cardiology, The Fourth Affiliated Hospital of School of Medicine, and International School of Medicine, International Institutes of Medicine, Zhejiang University, Yiwu, China; ^2^ Liangzhu Laboratory, Zhejiang University Medical Center, Hangzhou, China; ^3^ Department of Gynecological Oncology, Fudan University Shanghai Cancer Center, Shanghai, China

**Keywords:** diabetic cardiomyopathy, biomarkers, early diagnosis, diagnostic imaging, inflammatory mediator, speckle tracking echocardiography, sequencing

## Abstract

Diabetic cardiomyopathy (DCM) is one of the most prevalent and severe complications associated with diabetes mellitus (DM). The onset of DCM is insidious, with the symptoms being obvious only in the late stage. Consequently, the early diagnosis of DCM is a formidable challenge which significantly influences the treatment and prognosis of DCM. Thus, it becomes imperative to uncover innovative approaches to facilitate the prompt identification and diagnosis of DCM. On the traditional clinical side, we tend to use serum biomarkers as well as imaging as the most common means of diagnosing diseases because of their convenience as well as affordability. As we delve deeper into the mechanisms of DCM, a wide variety of biomarkers are becoming competitive diagnostic indicators. Meanwhile, the application of multiple imaging techniques has also made efforts to promote the diagnosis of DCM. Besides, the spurt in sequencing technology has made it possible to give hints about disease diagnosis from the genome as well as the transcriptome, making diagnosis less difficult, more sensitive, and more predictive. Overall, sequencing technology is expected to be the superior choice of plasma biomarkers for detecting lesions at an earlier stage than imaging, and its judicious utilization combined with imaging technologies will lead to a more sensitive diagnosis of DCM in the future. Therefore, this review meticulously consolidates the progress and utilization of various biomarkers, imaging methods, and sequencing technologies in the realm of DCM diagnosis, with the aim of furnishing novel theoretical foundation and guide future research endeavors towards enhancing the diagnostic and therapeutic landscape of DCM.

## 1 Introduction

Diabetes mellitus (DM) is a common metabolic disease featuring organ dysfunction caused directly or indirectly by hyperglycemia. It ranks among the most prevalent and rapidly increasing chronic diseases globally (1). It was estimated that nearly 10.5% adults, ranging from 20–79 years, were suffering from managing DM worldwide, with type 1 diabetes mellitus (T1DM) and type 2 diabetes mellitus (T2DM) constituting the major types, and T2DM accounting for 90% of cases (2–4). According to the fluctuations in disease prevalence, it has been calculated that without intervention, the number of people with DM will reach 783 million globally by 2045, representing 12.2% of the population (5). At the same time, the prevalence of T2DM, the mainstay of DM, is set to reach 700 million (2). The escalation imposes a heavy healthcare expenses burden on global healthcare system. DM patients are vulnerable to a spectrum of complications, including diabetic retinopathy, diabetic nephropathy, diabetic cardiomyopathy (DCM), and neurological issues (1, 6). Importantly, the prevalence of heart failure in DM patients is four-times higher than in the general populations, severely affecting their quality of life (7).

DCM stands out as a primary cause of heart failure in diabetic patients, specifically defined as cardiomyopathy developing in the absence of traditional cardiovascular risk factors such as coronary artery disease, valvular disease, or hypertension (8). Research categorizes DCM into two distinct phenotypes based on the ejection fraction (EF): the restrictive phenotype with preserved EF and the dilated phenotype with reduced EF (9). Initial symptoms of DCM often present as subclinical myocardial fibrosis and cardiac remodeling, primarily manifesting as early diastolic dysfunction (10). Moreover, recent hypotheses propose that early-stage DCM is characterized by a phase of myocardial hypercontractility triggered by metabolic imbalances, which progresses into a subclinical phase without overt symptoms (11). As the condition worsens, patients may develop symptoms like fatigue, palpitations, exertional dyspnea, arrhythmias, and angina pectoris, ultimately culminating in left ventricular (LV) failure and potentially life-threatening heart failure, alongside other diabetes-related complications (12). Although conventional imaging techniques like echocardiography can detect diastolic dysfunction in 40%–75% of T1DM or T2DM (13, 14), the insidious onset and subtle early symptoms of DCM can lead to missed early diagnosis or treatment. The current gold standard for diagnosing DCM is endomyocardial biopsy (EMB), which enables the detection of specific histological and ultrastructural changes in early-stage DCM. Histological examinations often reveal increased collagen volume, sparse coronary microvasculature, and advanced glycation end-product accumulation in the coronary microvasculature (9). However, due to its invasiveness and risks, EMB is predominantly confined to laboratory researches. Despite growing scholarly interest in DCM, existing guidelines lack well-defined and concrete diagnostic criteria for early stage of DCM.

Numerous studies have proposed potential diagnostic strategies for DCM, focusing on biomarkers, imaging techniques, and sequencing technologies. As for biomarkers, research mainly revolves around inflammatory mediators, fibrosis markers, antioxidant markers, cardiac hypertrophy markers and myocardial injury markers, which play essential roles in the pathogenesis of DCM (15). Researchers have mainly employed various imaging techniques to evaluate early cardiac changes in DCM, such as myocardial fibrosis, myocardial hypertrophy, and structural alterations in the diabetic myocardium. These imaging methods offer significant advantages over the invasive EMB in terms of non-invasiveness, reliability and repeatability (16, 17). Additionally, advancements in sequencing technologies showed great promise for the early diagnosis of DCM. Some studies have even leveraged sequencing technology to investigate the genetic, transcriptional and epigenetic underpinnings of DCM, opening new avenues for the diagnosis of DCM (18–20). This review consolidates the research progress on biomarkers, imaging methods, and sequencing technologies for DCM diagnosis, aiming to provide a comprehensive reference for the development of accurate and efficient diagnostic strategies and models.

## 2 Potential biomarkers for diabetic cardiomyopathy

### 2.1 Mediators of inflammation

**Figure 1:**
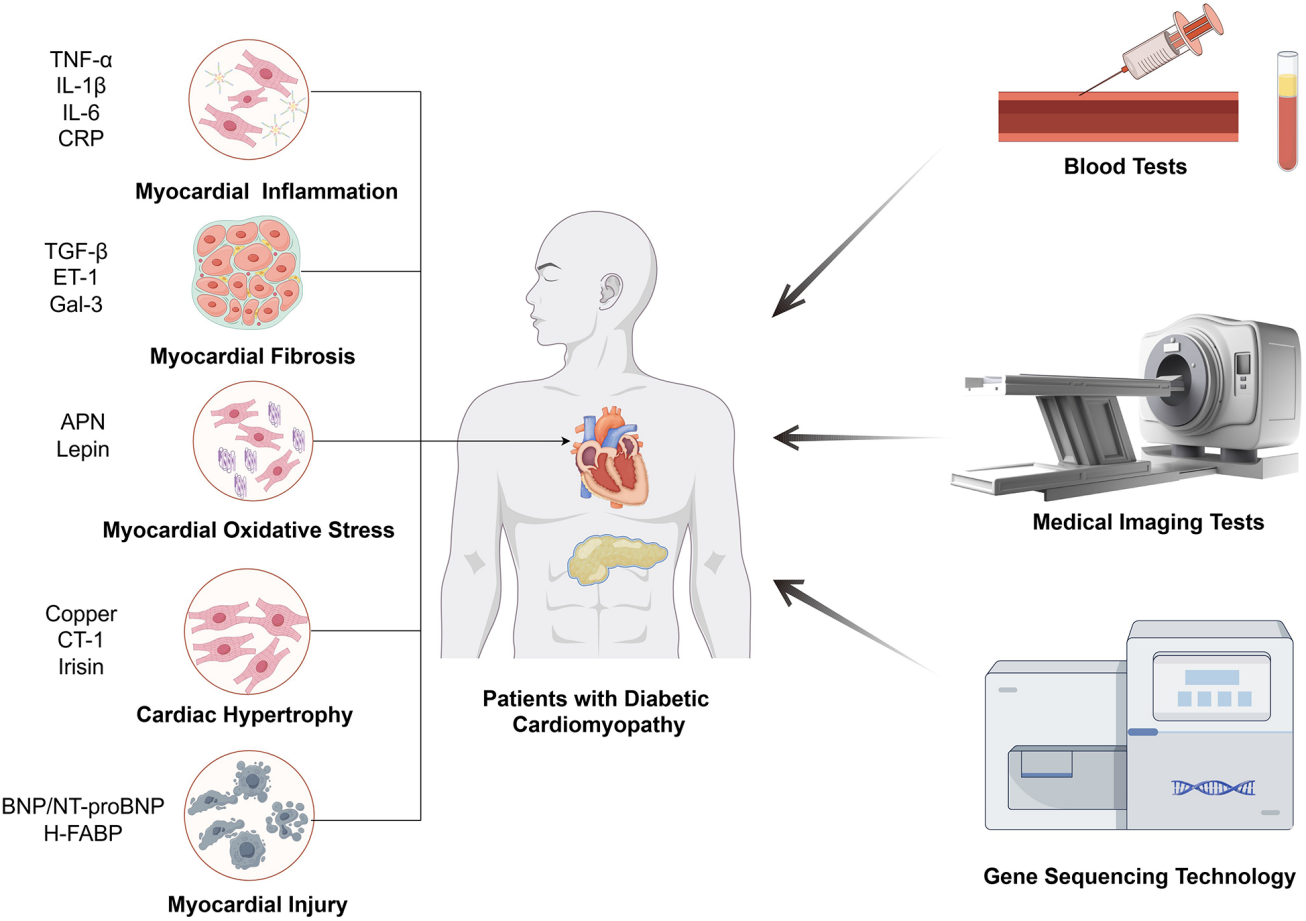
The main means of inspection for DCM. The biomarkers of DCM diagnosis through blood tests are related with five mechanisms, including myocardial inflammation, myocardial fibrosis, myocardial oxidative stress, cardiac hypertrophy, myocardial injury.

Persistent hyperglycemia stimulates inflammatory pathways, which are core mechanisms mediating myocardial dysfunction in DCM (21). It has been reported that cytokine and chemokine release induced by high blood glucose level would result in a common signaling pathway, known as nuclear factor kappa B (NF-κB) (22). Tumor necrosis factor alpha (TNF-α), interleukin (IL)-6, IL-1β and C-reactive protein (CRP) are all notable components of the downstream mediators, Figure 1 and Table 1. Elevated TNF-α levels have been observed in DCM patients (23, 24), which was particularly associated with LV diastolic dysfunction (25). IL-1β and IL-6 are also significantly elevated in both DCM animal models (27) and DCM patients (23) as compared to DM individuals, Figure 1 and Table 1. IL-6, in particular, has been significantly correlated with DCM incidence in long-term clinical studies (28, 29). Simultaneously, while CRP exacerbates LV dysfunction in DCM patients (31–36), its application as a diagnostic marker is hampered by its non-specificity and close association with infections or stressed states. Overall, inflammatory mediators are highly sensitive for the detection of DCM, especially for the assessment of DM populations at high pathogenic risk, but their specificity is susceptible to immune status.

**Table 1:** Potential biomarkers for diagnosing diabetic cardiomyopathy.

Biomarker	Indicative trends in plasma	Pathophysiological pathway	Reference
Inflammatory mediators			
TNF-α	Elevated	NF-κB signaling pathway	(23–26)
IL-1β/IL-6	Elevated	NF-κB signaling pathway	(23, 27–30)
CRP	Elevated	NF-κB signaling pathway	(31–36)
Fibrosis markers			
TGF-β	Elevated	TGF-β/Smads, MAPK and ERK1/2 signaling pathway	(37–42)
ET-1	Elevated	ET-1 dependent signaling pathway	(43)
Gal-3	Elevated	TLR4/MyD88/NF-κB signaling pathway	(44–46)
Antioxidant markers			
APN	Diminished	Activation of PPAR-γ	(47–50)
Leptin	Elevated	AMPK and COX2 dependent signaling pathways	(23, 51)
Myocardial hypertrophy markers			
Copper	Elevated	Copper dependent reactions	(52, 53)
CT-1	Elevated	JAK/STAT and MAPK pathways	(54, 55)
Irisin	Diminished	AMPK-mTOR signaling pathway and αV/β5-AKT signaling	(56)
Myocardial injury markers			
BNP	Elevated	PKG-STAT3 pathway	(57, 58)
NT-ProBNP	Elevated	Inhibiting pyroptosis pathway through P2X7 receptors	(57, 59, 60)
H-FABP	Diminished	MMP and JAK/STAT3 signaling pathways	(61, 62)

### 2.2 Fibrosis markers

Myocardial fibrosis is an essential stage in the process of progression of cardiomyocyte dysfunction, in which it is accompanied by the production of a number of specific factors, and the detection of changes in the levels of these factors can be applied as a basis for the diagnosis of myocardial fibrotic changes in DCM (63, 64). TGF-β is one of the most prominent profibrotic cytokines promoting extracellular matrix accumulation. Elevated plasma TGF-β levels have been observed in DCM patients, Figure 1 and Table 1 (23). And experiments have shown that high glucose could dysregulate TGF-β signaling through Smad3/Smad4 pathway in direct or indirect ways, which notably caused fibroblast activation and fibrosis in heart (64–66). In addition to TGF-β, there is a factor endothelin-1 (ET-1) that affects cardiomyocyte fibrosis independently of blood glucose. ET-1 is a profibrotic peptide produced by vascular endothelial cells in response to inflammation or oxidative stress. ET-1 could independently activate cardiac fibroblasts, promoting the development of pathologic myocardial fibrosis (43). Galectin-3 (Gal-3), a lectin family protein, is also linked to the fibrotic processes in cardiovascular diseases and is elevated in diabetic patients with mildly decreased EF and reduced global longitudinal strain (GLS), which was an early evidence of LV dysfunction for DCM, Figure 1 and Table 1 (44, 67, 68). Several studies have demonstrated that GLS was closely related to Gal-3, suggesting that Gal-3 combined with GLS served as predictors of early LV dysfunction in DCM (44, 45). In other diseases, Gal-3 expression was regulated by epigenetic mechanisms, and we look forward to further studies on the potential relationship between Gal-3 methylation levels and DCM (69, 70). Generally speaking, fibrosis markers are supposed to be combined with imaging techniques to obtain better sensitivity and specificity for DCM diagnosis.

### 2.3 Antioxidant markers

Studies have shown that DCM leads to alterations in the cardiomyocyte metabolome and related gene expression, resulting in hyperglycemia, cardiac lipid accumulation as well as oxidative stress (71, 72). Other research also linked mitochondrial dysfunction and oxidative stress to DCM (73). In these studies, oxidative stress is a direct influence on cardiac function that is ultimately shared by different pathways. Adiponectin (APN) and leptin are common substances that can alter oxidative stress by affecting metabolism, however, the former is a protective factor and the latter is harmful (71, 74). APN is an insulin-sensitizing hormone with levels inversely correlated to LV hypertrophy, Figure 1 and Table 1 (69). Zhao et al. noted that lower APN levels are linked with increased cardiovascular disease incidence (70). Studies have found that low plasma APN levels in DCM patients cause oxidative damage in cardiomyocytes (71). Similarly, Shaver et al. found that serum APN levels in DCM patients were lower than those in DM patients and markedly much lower than in healthy controls (12), indicating that clinical APN levels may serve as a monitoring tool for DCM patients. In terms of leptin, it is an adipose-derived hormone proportional to body fat, correlates with adverse cardiovascular outcomes, Figure 1 and Table 1 (47, 75). Higher serum leptin levels have been observed in DCM compared to DM controls (23), and were positively correlated with interventricular septum thickness *in vivo* (48). The mechanism could be explained by mitochondrial production of reactive oxygen species (ROS), which is the downstream effector of high leptin levels. And the relationship between development of diabetic vascular injury and leptin has been firmly strengthened through the pathogenic mechanism of oxidative stress (49). To summarize the clinical value of antioxidant markers, they can sensitively predict the development of cardiovascular outcomes, which are essential for the early prediction and diagnosis of DCM.

### 2.4 Cardiac hypertrophy markers

Cardiac hypertrophy is a common pathological change in many cardiac diseases such as atherosclerosis, heart failure and likewise for DCM. From pathophysiological aspect, cardiac hypertrophy is the result of an imbalance in energy metabolism with disruption of glucose or ionic homeostasis, which is one of the typical features of DCM (76). It has been proved that copper deficiency can induce cardiac hypertrophy and aggravate cardiomyopathy in DCM patients (52). And the defective excretion and uptake of copper are markedly altered throughout the body, where concentration of copper is elevated in plasma and extracellular myocardial cells but decreased in cardiac myocytes (53). Therefore, in combination with circulating copper levels as well as imaging findings, it can become possible to make a presumption as to whether DM patients are predisposed to cardiac hypertrophic lesions. In addition to copper, cardiotrophin-1 (CT-1), a member of the glycoprotein 130 family, is also a potent inducer of cardiac hypertrophy, Figure 1 and Table 1 (74). Several studies have demonstrated that CT-1 is highly expressed in DCM and other chronic heart disease, presenting a significant positive correlation between CT-1 plasma levels and LV mass index, which indicates its critical role in DCM pathogenesis through its involvement in myocardial remodeling (47, 48, 75). As for protective factors against cardiac hypertrophy, irisin is an important member with additional roles in regulating glucose metabolism. It mainly exerted protective effects through integrin αV/β5-AKT signaling and AMPK/mTOR signal pathway in DCM patients, Figure 1 and Table 1 (72, 73). For its rapid response to cardiac hypertrophy, irisin sheds light on early detection of DCM and pharmacological treatments. Taken together, the results of many studies have shown that cardiac hypertrophy markers reflect excellent sensitivity and specificity for the diagnosis of DCM, especially for early risk detection.

### 2.5 Myocardial injury markers

DCM is tightly linked to myocardial injury as well as heart failure, for chronic neurohormonal dysregulation and endothelial dysfunction create a milieu that predisposes individuals to more serious cardiac disease (77). For various DCM-associated disorders of the cardiovascular system, natriuretic peptides are an important family of biomarkers, especially brain natriuretic peptide (BNP) and its inactive precursor N-terminal pro-brain natriuretic peptide (NT-proBNP). BNP is primarily synthesized and secreted by the ventricles of the heart in response to overload volume or pressure, and exerts efforts in different target cells (78). The rich set of features include vasodilation, anti-inflammatory effects, anti-fibrotic effects, and antihypertrophic effects, which are supposed to relieve myocardial damage (79). Therefore, it mechanistically makes sense for BNP and NT-proBNP to contribute to the diagnosis of DCM. Researchers found that the combination of conventional echocardiography with BNP and NT-proBNP can effectively detect diastolic dysfunction (57, 59), which is often prior to symptomatic DCM and generally accompanied by subtle LV hypertrophy, Figure 1 and Table 1. However, normal natriuretic peptide levels can also be observed in some patients with abnormal diastolic function (57, 59), which limits the definitive diagnosis based solely on BNP levels. Apart from BNP or NT-proBNP, heat-fatty acid binding protein (H-FABP), a cytosolic lipoprotein that is abundant in myocardial tissues, also exhibits high specificity for myocardial injury, which is released early in response to cardiomyocyte damage. And serum H-FABP levels were significantly higher in DM patients compared to healthy controls (61), suggesting its promising role as a biomarker in asymptomatic DCM patients, Figure 1 and Table 1. Moreover, Shearer et al. established that reductions in plasma H-FABP may contribute to the correction of insulin resistance and glucose uptake in the heart (80), which underscores the necessity for further research into the relationship between H-FABP and DCM severity to enhance our understanding of its diagnostic role in early-stage DCM patients. Overall, myocardial injury markers were assessed to have good clinical value for myocardial injury and heart failure due to DCM, but the sensitivity and specificity for the early diagnosis of DCM remain to be supported by more evidence.

## 3 Diagnostic imaging methods for DCM

### 3.1 Echocardiography

**Figure 2:**
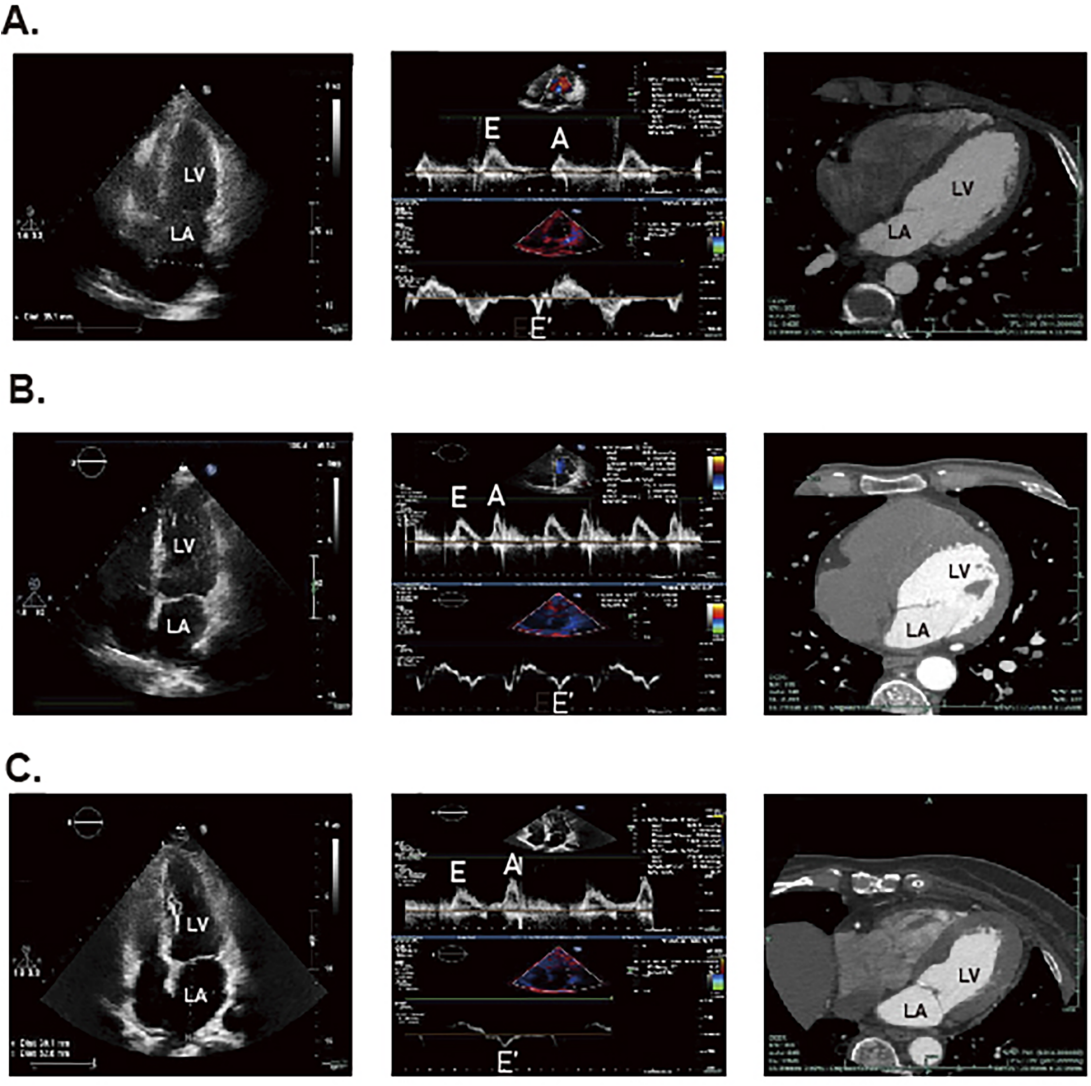
The STE and CT results for DCM patients, DM patients and healthy individuals. The panel **(A)** represents the result of healthy individuals. The panel **(B)** represents the result of patients with DM. The **(C)** panel represents the result of patients with DCM.

Conventional echocardiography (CE) can be used to assess intermediate-term DCM, which is mainly characterized by decreased E/A ratio, increased LV myocardial mass, decreased left ventricular diameter (LVD), and prolonged deceleration time, Figure 2 (81). Studies also showed that CE can identify abnormalities associated with LV hypertrophy and impaired diastolic filling present in early DCM (81, 82). In clinical practice, CE is non-invasive, painless, safe, affordable and available from clinics to general hospitals, which is widely regarded as the preferred method to evaluate DCM cardiac structures and function. And among all available imaging technologies, CE is the most cost-effective imaging modality, which leads to high frequency of CE utilization in patients with DCM. However, CE has several limitations, including operator subjectivity, angular correlation, and operator-induced noise. Additionally, the technique is susceptible to variations in patient heart rate and cardiac volumetric status. Furthermore, the pseudo-normal LV filling pattern observed in DM patients may also lead to underestimation of early DCM (83). Currently, there are several novel imaging techniques in echocardiography that are more helpful in workup of patients with DCM, however with higher expense. Tissue doppler imaging (TDI) serves as a valuable diagnostic imaging technique primarily utilized in the assessment of cardiovascular diseases. In patients with intermediate-term DCM, TDI is predominantly characterized by an E/E' ratio exceeding 15 (81). It enables the evaluation of myocardial velocities across various myocardial segments and effectively detects longitudinal, circumferential, and radial contractions of myocardial fibers, thereby quantifying the degree of ischemia and fibrosis. Di Bonito et al. discovered that 50% of diabetic patients exhibiting no cardiac dysfunction on CE examinations had an E/A ratio greater than 1 when assessed by TDI (84), which underscored the enhanced sensitivity of TDI compared to CE. However, the application of TDI is heavily influenced by angle, which makes it quite dependent on the technical competence of the professionals (85). Speckle tracking echocardiography (STE) is a relatively novel imaging modality for assessing myocardial tissue function. It overcomes limitations of traditional echocardiography by minimizing interference from factors such as examiner subjectivity, angle dependency and noise interference (86). STE provides progressive diastolic and systolic function assessment by tracking explicit speckle patterns, produced by interferences of ultrasound beams with the myocardial tissue. As a result, it facilitates the detection of myocardial deformation across three axes: radial, circumferential, and longitudinal strains (87). In the evaluation of systolic function in DCM patients, GLS is the most widely used parameter, indicating the degree of impairment. Reports showed that reduced GLS was hypersensitive to early cardiac lesions in DM and there was a correlation with the severity of symptoms (88–90). Additionally, another new STE marker, peak systolic longitudinal rotation, was also found to be diminished in early stages of DCM (91). Therefore, the new parameters, which have been gradually mined along with the development of STE, have injected new vigor into the early imaging diagnosis and assessing cardiac functional changes of DCM. Nevertheless, STE does face certain challenges, its accuracy is influenced by ventricular wall thinning and abnormal ventricular remodeling.

### 3.2 Cardiovascular magnetic resonance

The early manifestation observed via cardiovascular magnetic resonance (CMR) in DCM patients is characterized by altered cardiac metabolism (81). And CMR can detect early interstitial fibrotic changes in DCM, which could uniquely be detected by CMR-derived T1 mapping at present, whereas STE was only able to predict the risk of subsequent development of heart failure and thus detect further progression of fibrosis (92). Compared to CE, CMR provides superior spatial and temporal resolution for evaluating ventricular size, left ventricular ejection fraction (LVEF) and myocardial mass distribution (81). Furthermore, CMR provides information about myocardial ischemia and precise tissue characterization that could not be detected with echocardiography (93). DCM can also be classified into three primary stages based on CMR findings: the early stage, featuring slight LV hypertrophy that may be accompanied by diastolic insufficiency; the intermediate stage, exhibiting progressive cardiomyocyte hypertrophy, increased myocardial mass and thickening of the ventricular wall, which leads to diastolic dysfunction and mild systolic dysfunction (EF < 50%); and the late stage, characterized by further increases in LV size, wall thickness and mass, accompanied by evident diastolic and systolic dysfunction (94). Fischer et al. demonstrated that tagging MRI provides high-precision assessments of myocardial motion, facilitating the measurement of deformation during the cardiac cycle and enabling the monitoring of early DCM changes (95). However, the cost of CMR devices and the requirement for high image quality limit its application scenarios (93). In terms of patient costs, CMR does not have a dominant position among several imaging technologies, while its accessibility is only surpassed by CE. Besides, CMR has other limitations including lengthy duration of examination, potential claustrophobia for some patients, relatively poor sensitivity to diastolic dysfunction and incompatibility with certain pacemakers or implantable defibrillators.

### 3.3 Multi-slice computed tomography

The multi-slice computed tomography (MSCT) examination of advanced DCM predominantly reveals myocardial calcification associated with ischemia, Figure 2 (81). Notably, Schuijf JD et al. have demonstrated that MSCT can reliably assess LV dysfunction in T2DM patients (96). Additionally, it provides secure and reliable methods for patients who cannot undergo invasive coronary angiography or CMR, as well as for those with suspicious echocardiographic outcomes (97). For patients, although MSCT is second only to CE concerning examination expense, its diagnostic value is much lower than CMR as well as echocardiography. Nonetheless, most clinic guidelines firmly suggest high-risk patients with T2DM are supposed to undergo invasive coronary examination, which restricts the extensive application of MSCT in DCM (97). Moreover, MSCT has other notable limitations, including radiation exposure and the use of toxic contrast agents, which impede its position as the preferred diagnostic imaging modality. Also, the maintenance and site requirements of MSCT are more stringent than those of CE, making its application in DCM not as convenient as the latter. Therefore, further research is essential to optimize the use of contrast agents in MSCT and enhance its utility in clinical practice.

### 3.4 Nuclear imaging technology

Nuclear imaging is mainly applied to detect myocardial metabolism in the early stages of DCM and consists of two main techniques, gated single-photon emission computed tomography (G-SPECT) and positron emission tomography (PET) (81). Di Carli et al. have demonstrated that G-SPECT provides comprehensive data on ventricular function, myocardial wall thickness, exercise capacity, and diastolic parameters through three-dimensional imaging utilizing labeled myocardial perfusion agents (98). Given that the metabolism of tracers is influenced by metabolic alterations in disease states, G-SPECT presents considerable promise for the early detection of DCM (99). Similarly, PET is the most broadly applied radionuclide method to detect myocardial metabolism and to perform molecular imaging, due to its flexibility in radiotracer design and inherent quantitative capabilities (100, 101). It is rather helpful in patients who are obese or exhibit advanced LV dysfunction and coronary artery disease, conditions that may not be detected by G-SPECT imaging (102). Van den Brom et al. noted in early-stage DCM rat models that PET using ^18^F-2-fluoro-2-deoxy-D-glucose (^18^F-FDG) as a tracer revealed decreased myocardial glucose utilization and increased fatty acid oxidation (103). Overall, nuclear imaging technology offers specific advantages over CE with respect to resolution, reproducibility, and sensitivity. Nevertheless, the primary physical drawback of PET is low spatial resolution. However, it can be alleviated through technologies combination like PET/CT and PET/MR, which guarantee more precise localization of radiotracers (104). Other limitations include the cost of the radiotracer and the hazards of radiation to patients and technicians, and the requirements for equipment and space, making nuclear imaging technology still far from clinical diagnostic DCM.

## 4 Sequencing and its application in DCM diagnosis

Since the introduction of the dideoxy chain-termination method for DNA sequencing by Sanger et al. in 1976, sequencing technology has undergone remarkable advancements over the decades. Sanger sequencing translates nucleotide variations at each position into fluorescent signals by employing dideoxy-nucleoside triphosphates (ddNTPs) as chain terminators (105). Although, the efficiency of this method was subsequently enhanced through the use of polymerase chain reaction (PCR) and capillary electrophoresis, challenges related to sequence length, throughput and cost persisted.

**Figure 3:**
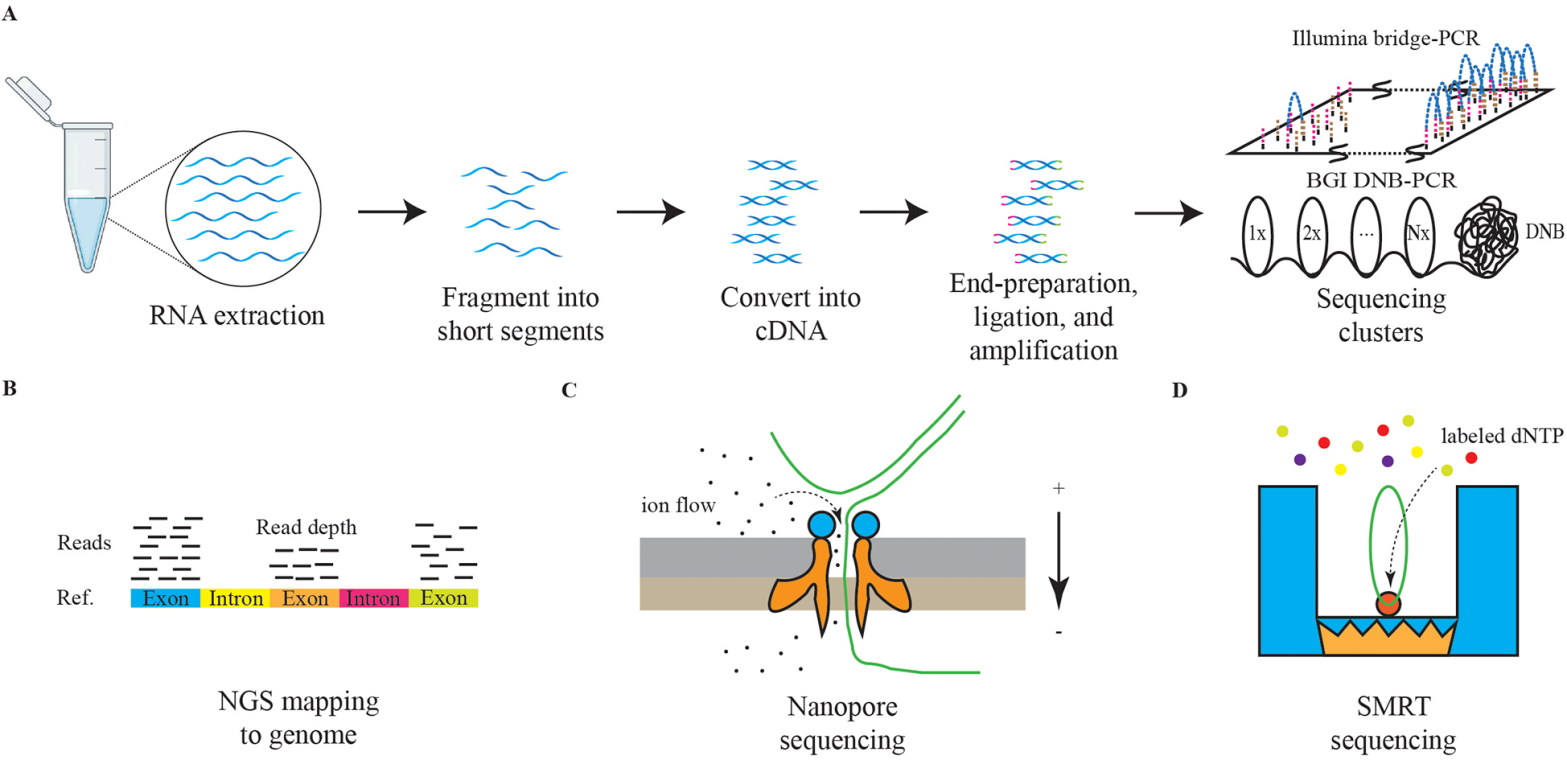
The schematic diagrams of NGS and TGS. The panel **(A)** represents the schematic diagram of NGS RNA sequencing. The graph **(B)** represents the schematic diagram of NGS reads mapping. The graph **(C)** represents the schematic diagram of nanopore sequencing. The graph **(D)** represents the schematic diagram of SMRT sequencing.

To overcome these limitations, next-generation sequencing (NGS) was developed. Rather than sequencing entire DNA molecules, NGS subdivides the total sequence into 150–200 base pairs (bp) small fragments, which are amplified using specialized PCR techniques, such as bridge PCR or rolling circle amplification (RCA), to improve the accuracy of basecalling (106, 107). Subsequently, the fragments are aligned to reconstruct a comprehensive genetic sequence, Figure 3 (108, 109). NGS significantly enhances sequencing throughput while lowering the barrier to the application of sequencing technologies, making it one of the most utilized methodologies in contemporary research and clinical settings (110). The resolution of both DNA and RNA through NGS has greatly advanced the understanding of human disease genomes and transcriptomes. In addition, technologies built upon NGS, including single-cell sequencing, high-throughput chromosome conformation capture (Hi-C), and assay for transposase-accessible chromatin using sequencing (ATAC-Seq), are being employed across a variety of diseases (111–113).

Currently, third-generation sequencing (TGS) technologies, characterized by long-read sequencing (LRS), are gaining traction. Methods such as nanopore sequencing and single-molecule real-time (SMRT) sequencing are providing deeper insights into the genetic transcription mechanisms underlying diseases, Figure 3 (114, 115). Furthermore, the advantages of TGS in interpreting methylation modifications are significantly enhancing our understanding of pathological processes (116).

**Figure 4:**
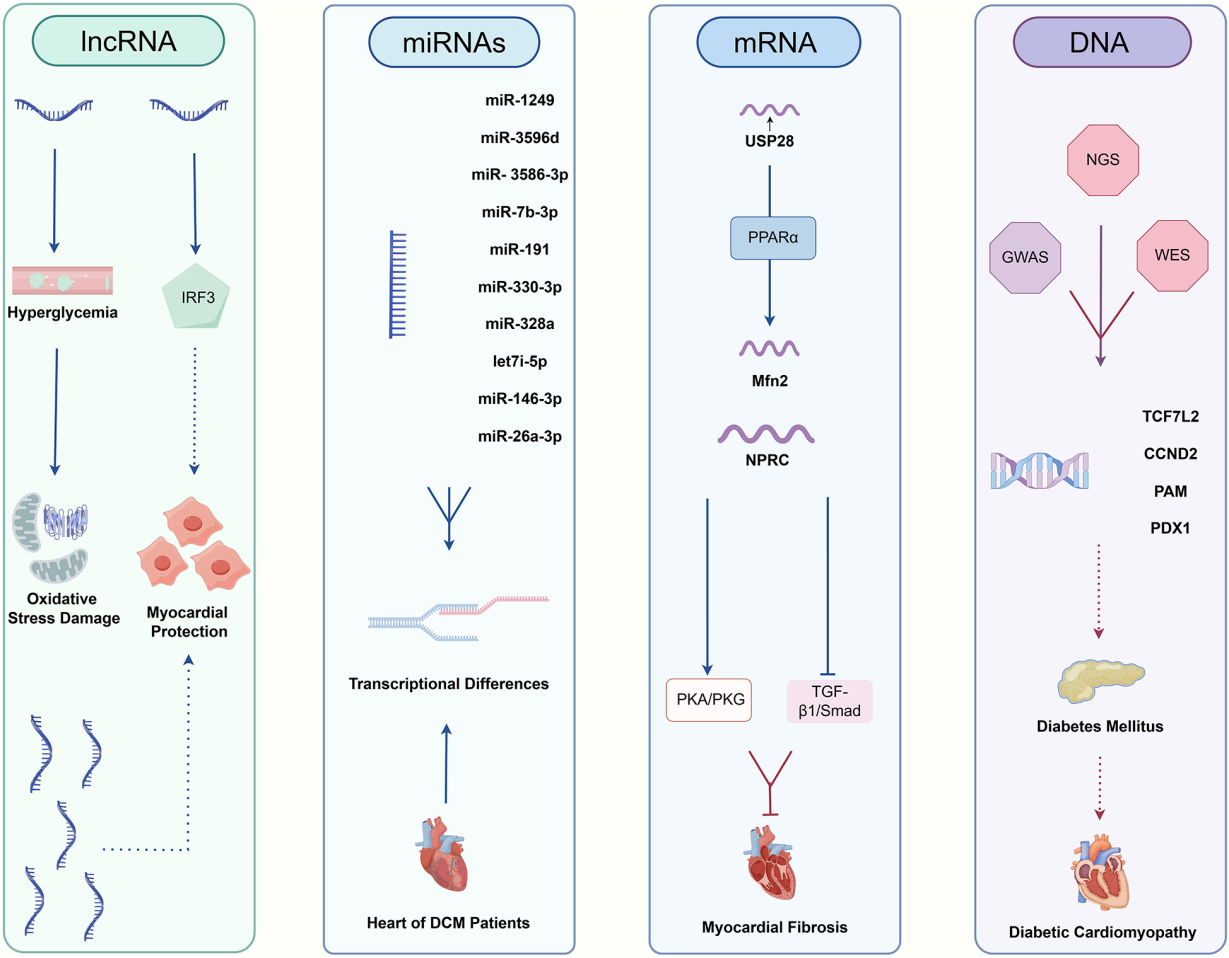
The relative pathways of non-coding RNAs and DNA taking part in DCM.

Sequencing technology is increasingly utilized in DCM, Figure 4. Among the diagnostic approaches, NGS technology for RNA has predominantly focused on research related to DCM, while applications of DNA sequencing and TGS remain comparatively limited. RNA sequencing technology has been instrumental in identifying numerous novel long non-coding RNAs (lncRNAs) associated with glycemic cardiopathy, which may prove useful for both diagnosis and monitoring disease progression. For instance, Yu et al. demonstrated that lncRNA NONRATT007560.2 regulates oxidative stress and apoptosis in cardiomyocytes induced by high glucose levels (117). Additionally, Xie et al. reported that lncRNA ZNF593-AS mitigates DCM by suppressing the IRF3 signaling pathway (118). A transcriptomic screening study using a rat model of DCM also identified five significant lncRNAs implicated in the condition (119). The above study relies on NGS technology to screen underscore the potential of RNA sequencing in uncovering diagnostic targets and elucidating injury mechanisms in DCM, starting from the transcriptome level.

Moreover, NGS has revealed interesting targets among other non-coding RNAs. Mathur et al. first reported the differential expression of various microRNAs (miR-1249, miR-3596d, miR-3586-3p, miR-7b-3p, miR-191, miR-330-3p, miR-328a, let-7i-5p, miR-146-3p, miR-26a-3p) in diabetes-induced cardiac cells, suggesting their potential as minimally invasive diagnostic biomarkers (120). Dong et al. identified five circular RNAs (circRNAs) involved in the pathogenesis of DCM, which may serve as promising biomarkers and therapeutic targets for early-stage intervention (121).

In the realm of mRNA, sequencing technology has broad applications as well. For example, Xie et al. found a positive correlation between USP28 mRNA levels and Mfn2 mRNA levels in hearts of diabetic patients, indicating that USP28 may promote PPARα nuclear translocation to influence Mfn2 transcription in cardiomyocytes (122). Similarly, Meng et al. demonstrated that NPRC deletion attenuates cardiac fibrosis in a diabetic mouse model by activating PKA/PKG signaling and inhibiting TGF-β1/Smad pathways, which represents an essential consideration for clinical diagnosis and treatment (123). Furthermore, RNA sequencing has established a stronger connection between DCM and inflammation as well as cell death (124, 125). A series of studies leveraging RNA sequencing have led to a more profound understanding of both classical and non-classical pathways associated with DCM, facilitating the identification of novel diagnostic targets.

In addition to routine RNA sequencing, other NGS applications are being explored for DCM diagnosis. Ju et al. combined m6A-specific methylated RNA identification with NGS to demonstrate that m6A modification patterns are altered in DCM, suggesting clinical relevance for diagnosis and treatment (126). Su et al. took full advantage of single-cell technologies, including scRNA-seq and scATAC-seq, revealing the pivotal roles of fibroblasts and endothelial cells playing in driving DM myocardial fibrosis and contributing to cardiac dysfunction (127). Advances in genome sequencing studies contribute significantly to our understanding of DM as a primary contributor to DCM. Previous research has established a clear link between DCM and DM, with the progression of DM exacerbating DCM symptoms (128). DNA sequencing in diabetic patients and genome-wide association studies (GWAS) have identified multiple genes associated with DM development, including *TCF7L2*, *CCND2*, *PAM*, and *PDX1* (129, 130). These findings further emphasize the potential value of genome sequencing technologies, such as whole-genome sequencing (WGS) and whole-exome sequencing (WES), in the context of DCM. Distinguished from NGS, TGS could demonstrate genomic variants at higher resolution, especially for >50 bp mutations and variants in repeated sequence regions (131, 132). In previous studies, it has been found that advanced glycation end products (AGEs) and the downstream receptor for advanced glycation end products (RAGE) can lead to increased oxidative stress and activation of pro-inflammatory pathways in diabetic complications, like DCM, promoting the damage to target organs (133, 134). Researchers from India obtained high accuracy polymorphisms for *RAGE* gene in DM patients, which include two novel ones in the intron and 3′UTR region respectively. Analysis of genotype distribution and allele frequencies combined with clinical manifestations circled several risky *RAGE* gene polymorphisms resulting in DM and its complications (135). Additionally, nanopore sequencing has significantly enhanced our ability to analyze genomic variants in patients with cardiomyopathy (136). Apart from the application of TGS in detecting DCM pathogenic gene polymorphisms, LRS also plays an excellent role in revealing different RNA isoforms. Researchers found that RNA binding fox-1 homolog 2 (RBFOX2) plays a key role in the pathogenesis of DCM by mediating alternative splicing (AS) (137). By virtue of nanopore sequencing technology, a novel sequence of full-length tropomyosin 1 (TPM1) isoforms has been revealed, which was closely associated with chronic heart disease, including DCM, under RBFOX2-mediated AS regulation (138).

Challenges persist in the translation of sequencing technology into clinical diagnostics, particularly regarding the validation of biomarkers across broader populations and the reduction of associated costs. Conducting large-scale studies and pilot sampling in healthcare settings can help establish standardized processes, thereby improving the early detection and treatment of DCM. Overall, ongoing advancements in the integration of traditional clinical practices with sequencing technologies hold significant potential to revolutionize the management of diseases at their early stages.

## 5 Concluding remarks

The elusive onset and atypical clinical manifestations of DCM pose significant challenges to early detection, resulting in the loss of opportunities for optimal therapeutic intervention. Traditional diagnostic methods such as EMB, is difficult to become a universal diagnostic method due to the invasiveness of the procedure, limiting their widespread applicability in early DCM patients. In recent years, with the deepening of research into the pathophysiological mechanisms of DCM, a range of novel biomarkers have emerged, showing promising potential in the diagnosis of this condition. Nevertheless, the clinical application of these biomarkers still confronts numerous challenges, including suboptimal diagnostic specificity and sensitivity due to various confounding factors. Consequently, there is an urgent and critical need for large-scale, systematic patient cohort studies to thoroughly explore the clinical utility and appropriate scope of these biomarkers.

Concurrently, emerging imaging modalities, exemplified by STE, CMR and PET have offered novel approaches for early DCM detection. These techniques enable precise capture of subtle signs of myocardial metabolic changes, providing robust imaging evidence for identifying early DCM patients. However, their high costs and the potential toxicity of contrast agents involved in some of these techniques remain as bottlenecks hindering their widespread adoption. In the future, with continuous technological advancements and effective cost control, these imaging approaches are expected to play an even more pivotal role in the early diagnosis of DCM.

Compared to the above more clinically relevant tests, sequencing technology is still some distance away from the application of DCM for clinical diagnosis. However, with the booming development of sequencing technology, its application field has gradually expanded from Mendelian genetic diseases to monogenic and polygenic genetic diseases. In terms of DCM, principally, NGS and TGS can not only detect DM and cardiomyopathy-related risk gene variants at the genomic level, but also dynamically monitor the process of cardiac damage, fibrosis, and cell death pathways at the transcriptomic level. This will enable us to achieve the aim of early prevention, diagnosis and treatment of DCM, significantly improving patient course. Currently, the translation of sequencing technology to DCM clinical diagnostics still needs to overcome the following urgent challenges, calling for more advances in relevant research directions. Firstly, although emerging DCM studies with different sequencing technology discover new massages from genomic or transcriptomic dimension, there is an urgent need for guidelines to clarify the scenarios in which sequencing can be used and the standard process for diagnosis. Secondly, the cost of sequencing is still expensive, especially for WGS, which limits it to the lab and makes it difficult to move into the clinic broadly like biomarkers or imaging technologies. There still exists room to develop new materials and technologies to achieve lower costs. Thirdly, we have witnessed various advanced algorithms created for analysis of sequencing data. However, there are certain programming thresholds for clinicians, such as, the ability of basic programming and the construction of dependent environments. We need some pipelines or software packages to break down barriers to sequence analysis.

Once these challenges are effectively addressed, the emerging application of sequencing will surely enhance human health protection and pave the road for a further boom in clinic research on DCM-like diseases. For an era of healthcare characterized by the development of precision medicine and personalized medicine, the joint advancement of traditional clinical examination and sequencing technology will open up new ways for prevention and diagnosis of multiple diseases.

## Glossary

2D-STE: two-dimensional speckle tracking echocardiography
3D-STE: three-dimensional speckle tracking echocardiography
AGEs: advanced glycation end products
AS: alternative splicing
AMPK: AMP-activated protein kinase
APN: adiponectin
ATAC-Seq: assay for transposase-accessible chromatin using sequencing
BNP: brain natriuretic peptide
bp: base pairs
CCND2: cyclin D2
CE: conventional echocardiography
circRNAs: circular RNAs
CMR: cardiovascular magnetic resonance
CRP: C-reactive protein
CT-1: Cardiotrophin-1
DCM: diabetic cardiomyopathy
ddNTPs: dideoxy-nucleoside triphosphates
DM: diabetes mellitus
EF: ejection fraction
EMB: endomyocardial biopsy
ET-1: endothelin-1
Gal-3: galectin-3
GLS: global longitudinal strain
G-SPECT: gated single-photon emission computed tomography
GWAS: genome-wide association studies
H-FABP: heat-fatty acid binding protein
Hi-C: high-throughput chromosome conformation
IL-1: interleukin-1
IL-1β: interleukin-1β
IL-6: interleukin-6
IRF3: interferon regulatory factor 3
lncRNAs: long ncRNAs
LRS: long-read sequencing
LV: left ventricular
LVD: left ventricular diameter
LVEF: left ventricular ejection fraction
m6A: N6-methyladenosine
Mfn2: mitofusin 2
MSCT: multi-slice computed tomography
mTOR: mammalian target of rapamycin
NF-κB: nuclear factor kappa B
NGS: next-generation sequencing
NPRC: natriuretic peptide receptor C
NT-ProBNP: N-terminal pro-brain natriuretic peptide
PAM: peptidylglycine α-amidating monooxygenase
PCR: polymerase chain reaction
PDX1: pancreatic duodenal homeobox 1
PET: positron emission tomography
PKA: protein kinase A
PKG: protein kinase G
RAGE: receptor for advanced glycation end products
RCA: rolling circle amplification
RBFOX2: RNA binding fox-1 homolog 2
ROS: reactive oxygen species
SMAD: small mother against decapentaplegic
SMRT: single-molecule real-time
sST2: soluble growth stimulation expressed gene2
STE: speckle tracking echocardiography
T1DM: type 1 diabetes mellitus
T2DM: type 2 diabetes mellitus
TCF7L2: transcription factor 7-like 2
TDI: tissue doppler imaging
TGF-β: transforming growth factor beta
TGS: third-generation sequencing
TNF-α: tumor necrosis factor alpha
TPM1: tropomyosin 1
T2DM: type 2 diabetes mellitus
WES: whole-exome sequencing
WGS: whole-genome sequencing
